# Genome-wide analysis and expression of the aquaporin gene family in *Avena sativa* L.

**DOI:** 10.3389/fpls.2023.1305299

**Published:** 2024-01-19

**Authors:** Xinyue Zhou, Dengxia Yi, Lin Ma, Xuemin Wang

**Affiliations:** Institute of Animal Sciences, Chinese Academy of Agricultural Sciences, Beijing, China

**Keywords:** *Avena sativa* L., aquaporin, gene family, genome-wide, abiotic stress

## Abstract

**Background:**

Oat (*Avena sativa* L.) belongs to the early maturity grass subfamily of the Gramineae subfamily oats (Avena) and has excellent characteristics, such as tolerance to barrenness, salt, cold, and drought. Aquaporin (AQP) proteins belong to the major intrinsic protein (MIP) superfamily, are widely involved in plant growth and development, and play an important role in abiotic stress responses. To date, previous studies have not identified or analyzed the *AsAQP* gene family system, and functional studies of oat AQP genes in response to drought, cold, and salt stress have not been performed.

**Methods:**

In this study, AQP genes (*AsAQP*) were identified from the oat genome, and various bioinformatics data on the AQP gene family, gene structure, gene replication, promoters and regulatory networks were analyzed. Quantitative real-time PCR technology was used to verify the expression patterns of the AQP gene family in different oat tissues under different abiotic stresses.

**Results:**

In this study, a total of 45 AQP genes (*AsAQP*) were identified from the oat reference genome. According to a phylogenetic analysis, 45 *AsAQP* were divided into 4 subfamilies (PIP, SIP, NIP, and TIP). Among the 45 *AsAQP*, 23 proteins had interactions, and among these, 5AG0000633.1 had the largest number of interacting proteins. The 20 *AsAQP* genes were expressed in all tissues, and their expression varied greatly among different tissues and organs. All 20 *AsAQP* genes responded to salt, drought and cold stress. The NIP subfamily 6Ag0000836.1 gene was significantly upregulated under different abiotic stresses and could be further verified as a key candidate gene.

**Conclusion:**

The findings of this study provide a comprehensive list of members and their sequence characteristics of the *AsAQP* protein family, laying a solid theoretical foundation for further functional analysis of *AsAQP* in oats. This research also offers valuable reference for the creation of stress-tolerant oat varieties through genetic engineering techniques.

## 1 Introduction

Oat (*Avena sativa* L.) belongs to the early-maturity grass subfamily of the Gramineae subfamily oats (Avena) and is an annual grain feeding crop of the genus *A. sativa* in the family of Gramineae. According to the seed type, oats can be divided into skin oats and naked oats. Oats exhibits barrenness, resistance to salinity, cold and drought, and a high grass yield, is rich in nutrients, has high palatability and other excellent characteristics, and is widely distributed in 42 countries and regions around the globe (Zhou et al., 1999; Wu et al., 2019; Ye et al., 2023; Zhang et al., 2023). *A. sativa* also plays an important role in promoting the development of grassland animal husbandry and livestock feeding in winter and spring and provides an ecological pasture for soil salinization and desertification control (Gang and Zhang, 2020). The cultivation of *A. sativa* is important for restoring degraded natural pastures and improving the utilization of saline soils and can also play a unique role in meeting the challenges of food security and promoting grassland animal husbandry and ecological environmental protection (Nan et al., 2017; Shi et al., 2023).

Aquaporins (AQPs) are a class of small molecular intrinsic membrane proteins with major intrinsic protein domains (MIPs) located on the cell membrane. Their primary function is to form “pores” on the cell membrane, allowing for the controlled movement of water in and out of cells, forming narrow channels that facilitate single-file transport. This enables signal transduction within plant cells and regulates the dynamic balance of water, promoting rapid transmembrane water transport (Wu et al., 2015; Afzal et al., 2016; Singh et al., 2020). The AQP family was first discovered in humans and then widely isolated in animals, plants, fungi, and bacteria (Hu et al., 2015; Feng et al., 2018; Zhang et al., 2022). Since the isolation of the first plant water channel protein *AtTIP*1 in *Arabidopsis thaliana* (Zhang and Du, 2014), an increasing number of AQP have been found in plants, such as *Zea mays* L., *Oryza sativa* Linn. and *Lycopersicon esculentum* Miller (Chaumont et al., 2001; Sakurai et al., 2005; Li et al., 2018). Based on structural features, plant AQP proteins can be categorized into seven subfamilies: plasma membrane intrinsic proteins (PIPs), vesicular membrane intrinsic proteins (tonoplast intrinsic proteins, TIPs), Nod26-like membrane intrinsic proteins (NIPs), small basic intrinsic proteins (SIPs), X intrinsic proteins (XIPs), GlpF-like intrinsic proteins (GIPs),.and hybrid intrinsic proteins (HIPs) (Bienert and Chaumont, 2011; Abascal et al., 2014; Kapilan et al., 2018). GIPs and HIPs are currently found only in mosses, ferns and algae (Danielson and Johanson, 2008; Anderberg et al., 2012; Maurel et al., 2015). Seven subfamilies have different functions and subcellular localizations. Specifically, PIPs, XIPs, SIPs, and some NIPs are usually localized in plasma membranes, and TIPs are mostly located in vesicular membranes (Takano et al., 2006; Maeshima and Ishikawa, 2008; Bienert et al., 2011). NIPs can also localize to the membrane of the ER (Diehn et al., 2015). All AQP have a conserved hourglass structure, which comprises six transmembrane (TM) helices (TM1-TM6) formed by six α-helix bundles and five loops (Loops A-E) (TÖrnroth-Horsefield et al., 2006). Plant AQP can not only mediate the transport of water between plant tissues but also act as an ion-selective permeable membranes to effectively mediate the transmembrane transport of other small molecules, nutrient elements, and metal ions, among others, in plants and effectively improve the stress tolerance of plant by affecting the operation of small-molecule solutes such as ions, gases, and heavy metals in the plant body (Yuan, 2022). The overexpression of *AtTIP*5;1 in *A. thaliana* enhances the tolerance to boron toxicity (Pang et al., 2010); Mutations in *AtNIP*3;1 or *AtNIP*7;1 improve the tolerance to arsenate toxicity (Xu et al., 2015; Lindsay and Maathuis, 2016); and *AtPIP*1;2 can mediate CO_2_ transport (Boudichevskaia et al., 2015).

Abiotic stresses such as salinity, drought, flooding, and high- and low- temperature stresses exert inhibitory effects on plant growth, and plants can only survive under adversity conditions, especially in widespread saline and arid environments, by resisting the damage caused by water deficit through the formation of various proteins, such as AQP to evolve specific physiological mechanisms adapted to adversity stresses (Hu et al., 2012; Zhang et al., 2013). Many studies have demonstrated that AQP proteins are not only widely involved in plant growth and development but can also respond to multiple abiotic stresses. *GmPIP*1:6 could improve the salt resistance of *Glycine max* (Li et al., 2015). In barley leaves, the expression of the *HvPIP*1;6 gene increased under salt stress conditions, promoting leaf growth (Fricke et al., 2006). The overexpression of rice *OsPIP*1-1 or *OSPIP*2-2 in *A. thaliana* significantly improves the drought tolerance of the plant (Guo et al., 2006), and the overexpression of *OsPIP*2 in rice can improve the survival rate of rice under low-temperature stress (Li et al., 2008). Soybean *GmTIP*1-1 enhances soybean resistance to abiotic stress through its interaction with GmSNARE proteins involved in abiotic stress regulation and stress-responsive protein GmF-box (Li et al., 2019). The ectopic overexpression of *TsTIP*1;2 in *A. thaliana* resulted in a significant enhancement of plant tolerance to drought, salt, and oxidative stresses (Wang et al., 2014). Overexpression of the *PvPIP*2;9 gene in *Panicum virgatum* resulted in increased plant height, leaf length, aboveground biomass, cellulose content, and protein content under drought stress (Zhang et al., 2020).

To date, no study has identified or analyzed the *AsAQP* gene family system, and functional studies of *A. sativa* AQP genes in response to drought, cold and salt stress have not been conducted. In this study, we performed a gene-wide identification of the *AsAQP* family using bioinformatics methods and analyzed the phylogenetic relationship among *AsAQP*, their gene structure, and their chromosome distribution through a covariance analysis and an analysis of the protein interaction network of AsAQP. A quantitative real-time PCR (qRT-PCR) analysis was performed to examine the gene expression patterns of *AsAQP* in different tissues and their responses to cold, drought and salt stress. An overall expression analysis of *A. sativa* revealed the role of the AQP members in the different biological processes of *A. sativa* and these findings lay the foundation for further research on the biological functions of the family members and provides a theoretical basis for a functional analysis of the AQP proteins under abiotic stresses.

## 2 Materials and methods

### 2.1 Screening and characterization of AQP family members in *A. sativa*

Whole genome files of *A. sativa* (GCA_022788535) were downloaded from the Ensembl Plants website (https://plants.ensembl.org/index.html, accessed on 19 April 2023). The sequences of *A. thaliana* AQP family proteins were downloaded from the TAIR database (https://www.arabidopsis.org/, accessed on 10 May 2023), a hidden Markov model (HMM) of AQP was downloaded from the Pfam database (https://pfam.xfam.org/, accessed on 12 May 2023), and the sequences of 35 A*. thaliana* AQP proteins were used as templates for comparison with the *A. sativa* genome using TBtools software, with an e-value of 10. The identified genes were screened, duplicates and shorter sequences were removed, and the candidate genes obtained from the preliminary identification were validated against the candidate AQP protein sequences through the online website NCBI Conserved Domain Search online website (https://www.ncbi.nlm.nih.gov/structure/cdd/wrpsb.cgi, accessed on 14 May 2023). The conserved structural domains of the candidate AQP protein sequences were validated, and the validation results were visualized using the software TBtools to eliminate redundant repeats and incomplete sequences that did not contain the MIP structural domains with the aim of obtaining all candidate genes of the *A. sativa* AQP gene family.

### 2.2 Evolutionary analysis of the AsAQP system

The AQP protein sequences of *A. sativa* were compared with those of wheat and *A. thaliana* using the ClustalW algorithm, and the phylogenetic tree was constructed with MEGA 7.0 software using the neighboring method. Based on the results of phylogenetic analysis, AsAQP were categorized into four classes: PIP, TIP, NIP and SIP.

### 2.3 Analysis of the *AsAQP* gene structure and conserved protein motifs

The gene structure of the *A. sativa* AQP family members was analyzed using the online tool MEME (http://meme-suite.org/tools/meme) and the *A. sativa* genome annotation file, and their conserved motifs were analyzed based on a predicted value of 10. TBtools was used to visualize and map the obtained gene structure and the distribution of introns and exons, and the conserved motif information was visualized and mapped.

### 2.4 Chromosomal localization and cis-acting element prediction of *A. sativa* AQP family genes

Chromosomal positions of *A. sativa* AQP family genes were mapped using TBtools software, and 2000 bp upstream of the start codon was extracted as the promoter sequence, and cis-acting element prediction of the promoter was performed using Plantcare.

### 2.5 Covariate gene analysis and gene amplification patterns

To further analyze the *A. sativa* AQP family genes and to investigate whether the *A. sativa* AQP genes contain tandem duplications or segmental duplications, the duplication events of the AQP genes were analyzed using MCScanX software. To investigate the replication relationship between the *A. sativa* AQP gene family and other species, the covariance of *A. thaliana*, *T. aestivum*, and *Z. mays* with the *A. sativa* AQP gene were analyzed using the One Step MCScanX module of TBtools software. Chromosome distribution maps of the covariance of *A. sativa* AQP were plotted using Advanced Circos of TBtools software.

### 2.6 Gene regulatory network analysis of the AsAQP

The AsAQP protein sequences were uploaded using the online software STRING (https://string-db.org/, accessed on 20 July 2023). Using the *A. thaliana* database sequence as reference, the AsAQP interacting proteins were predicted according to the known interactions between *A. thaliana* AQP proteins. Optimizing protein-protein interaction networks with Cytoscape 3.7.2 (Doncheva et al., 2019).

### 2.7 Plant materials and treatments

The oat variety selected in this experiment was *Avena sativa* cv. Mengyan No.1. Seeds with full grains were selected, cultivated in a petri dish until spouting, and then transplanted into a seedling cup at a vermiculite- to- nutrient soil ratio of 1:1, with 9 seeds per cup, and cultured in a greenhouse (day/night 25°C/18°C, 65%~75% relative humidity). The light intensity was 200-250 μmol·m^-2^s^-2^ and the seedlings were thinned once they grew to two-leaf monophase. After 14 days, each cup were subjected to stress. The plants in the cold treatment group were incubated in an incubator at 4°C, whereas those in the control group were cultured normally in a long-day incubator. The seedlings in the cold treatment group were sampled after 6 h, 12 h, 24 h and 48 h of treatment, and the untreated seedlings were also sampled as controls. In this study, 20% PEG6000 was used to simulate drought stress, and samples were collected at 6 time points (0 h, 2 h, 4 h, 8 h, 12 h and 24 h) during PEG6000 treatment. Moreover, 150 mM NaCl was used to simulate salt stress, and samples were collected at 6 time points (0 h, 1 h, 3 h, 6 h, 12 h and 24 h) during this treatment. Different tissue parts of *A. sativa* growing to the waxy ripening stage were sampled. Three biological replicates, each of which consisted of three single plants, were included in this study, and the samples (all of the germinated seedlings) were immediately frozen in liquid nitrogen and stored at -80°C until used for RNA extraction.

### 2.8 Gene expression analysis by qRT-PCR and statistical analyses

The FastPure Plant Total RNA Isolation Kit (TIANGEN, Beijing, China) was used for the isolation of total RNA. The RNA quality was assessed using the NanoDrop™ One/OneC microvolume UV-Vis spectrophotometer (Thermo Fisher Scientific, Waltham, MA, USA) and checked using RNAse-free 1% (w/v) agarose gel electrophoresis. cDNA was then synthesized using a TransScript II One-Step gDNA Removal and cDNA Synthesis SuperMix kit (Vazyme, Nanjing, China). The qRT-PCR primers were designed based on the full-length coding sequences (CDS) of 20 *AsAQP* genes, with *A. sativa AsActin* (KP257585.1) gene used as the reference gene (Xiang et al., 2020) (**Table S1**). The expression levels of 20 *AsAQP* genes in different tissues and organs and under drought, salt and cold stress were measured by qRT-PCR, which was performed using a Light Cycle96 with TB Green^®^ Premix Ex Taq™ II (TAKARA, Beijing, China).The thermal cycle program was as follows: an initial step at 95°C for 3 min, followed by 40 cycles of 95°C for 15 s and 60°C for 30 s. Three biological replicates of each response were included in the experiment, and the data were analyzed using the 2-^ΔΔCT^ method. The expression heatmaps of 20 genes in different tissues and organs and under different abiotic stresses were established by using TBtools software. Detailed qpcr data for different abiotic stress treatments in oats are available in (**Table S2**).

## 3 Results

### 3.1 Identification of AQP family genes in *A. sativa*

Characterization of the structural domain integrity of the AQP family genes identified by the hidden Markov model resulted in the identification of 45 family members, which is a higher number than the found in *A. thaliana*, which is consistent with the fact that the genome of *A. sativa* is larger than that of *A. thaliana*. The amino acid number of *A. sativa* AQP family members ranged from 176 to 324, the molecular weight of the proteins ranged from 18.84 kD to 34.93 kD, and the isoelectric point size was in the range of 5.33 to 11.12. Most of the *A. sativa* AQP members had 5-6 transmembrane structural domains, but AVESA.00001b.r3. 6Cg0003382.4 contained only three transmembrane structural domains, probably because the other transmembrane structural domains were lost during evolution (**Table 1**).

**Table 1:** Characteristics of AQP family.

Gene ID	Chr Location	Amino acid number	Molecular Weight	PI	Number of domains	Subcellular Location
AVESA.00001b.r3.1Ag0000163.4	1A:18777595:18781251	176	18837.18	6.8	5	Cytoplasm
AVESA.00001b.r3.1Ag0000210.1	1A:26075839:26077471	284	29616.44	6.82	6	Cytoplasm
AVESA.00001b.r3.1Ag0000611.1	1A:219000192:219001574	265	27844.26	8.13	6	Cytoplasm
AVESA.00001b.r3.1Ag0000703.1	1A:235675639:235694181	288	29998.93	9.44	6	Cytoplasm
AVESA.00001b.r3.1Cg0001352.1	1C:359258801:359275555	298	30973.1	9.27	6	Cytoplasm
AVESA.00001b.r3.1Dg0000137.3	1D:15481990:15485492	220	23204.23	9.84	5	Cytoplasm
AVESA.00001b.r3.1Dg0000683.1	1D:226471727:226483570	275	28584.1	9	5	Cytoplasm
AVESA.00001b.r3.1Dg0003363.2	1D:460369178:460370960	249	25694.82	5.64	6	Cytoplasm
AVESA.00001b.r3.2Ag0001610.3	2A:354314028:354316192	273	28671.16	6.99	5	Cytoplasm
AVESA.00001b.r3.2Ag0001780.4	2A:365283513:365285450	191	20580.86	9.84	3	Endoplasmic reticulum
AVESA.00001b.r3.2Cg0002326.20	2C:506486769:506489056	284	30125.98	8.29	5	Cytoplasm
AVESA.00001b.r3.2Dg0001533.1	2D:149839172:149841260	289	30340	7.68	6	Cytoplasm
AVESA.00001b.r3.2Dg0001667.1	2D:157523494:157524720	248	25084.08	5.53	6	Vacuoles
AVESA.00001b.r3.2Dg0001704.2	2D:160723843:160725761	271	28740.25	8.29	5	Cytoplasm
AVESA.00001b.r3.3Ag0000885.1	3A:80090114:80091108	287	29344.04	8.03	6	Cytoplasm
AVESA.00001b.r3.3Ag0000886.1	3A:80150994:80153138	248	25144.36	6.69	6	Vacuoles
AVESA.00001b.r3.3Cg0000766.1	3C:77173484:77174927	267	28115.3	6.41	6	Cytoplasm
AVESA.00001b.r3.3Cg0001892.1	3C:488409080:488411280	249	25188.38	6.69	6	Cytoplasm
AVESA.00001b.r3.3Dg0000336.1	3D:31574338:31575351	251	25373.7	6.69	6	Vacuoles
AVESA.00001b.r3.4Ag0003826.1	4A:456822381:456823599	282	28927.44	6.39	6	Cytoplasm
AVESA.00001b.r3.4Dg0000045.1	4D:10680842:10683728	313	33262.78	8.09	6	Vacuoles
AVESA.00001b.r3.4Dg0000047.3	4D:10778942:10781137	285	30145.49	9.69	6	Cytoplasm
AVESA.00001b.r3.4Dg0003906.1	4D:452129830:452131114	278	28427.89	6.39	6	Cytoplasm
AVESA.00001b.r3.5Ag0000631.1	5A:50923418:50926380	247	26272.27	9.92	5	Cytoplasm
AVESA.00001b.r3.5Cg0002672.1	5C:555466994:555468235	307	32005.23	9.08	6	Cytoplasm
AVESA.00001b.r3.5Dg0003115.1	5D:466973272:466974714	283	29911.72	9.16	6	Cytoplasm
AVESA.00001b.r3.6Ag0000836.1	6A:196893406:196897053	210	22411.94	7.83	5	Cytoplasm
AVESA.00001b.r3.6Ag0001241.1	6A:264445895:264448870	289	30749.71	9	6	Cytoplasm
AVESA.00001b.r3.6Ag0001372.1	6A:278089789:278092805	283	30119.83	7.66	5	Cytoplasm
AVESA.00001b.r3.6Ag0001373.5	6A:278105380:278108492	206	21831.17	6.81	4	Cytoplasm
AVESA.00001b.r3.6Ag0002125.1	6A:333125232:333127028	290	30787.64	8.61	6	Cytoplasm
AVESA.00001b.r3.6Cg0001137.1	6C:109271640:109274280	281	30015.55	8.81	5	Cytoplasm
AVESA.00001b.r3.6Cg0002765.1	6C:461327976:461331820	324	34929.28	7.29	6	Cytoplasm
AVESA.00001b.r3.6Cg0003240.1	6C:571756493:571759040	317	33562.83	11.12	5	Cytoplasm
AVESA.00001b.r3.6Cg0003268.1	6C:578068589:578069781	249	25191.23	5.88	6	Vacuoles
AVESA.00001b.r3.6Cg0003380.2	6C:594963353:594967184	241	25654.36	5.91	4	Cytoplasm
AVESA.00001b.r3.6Cg0003382.4	6C:595022908:595026522	183	19547.86	9.2	3	Cytoplasm
AVESA.00001b.r3.6Dg0000172.1	6D:32971223:32973517	280	29598.32	9.17	6	Cytoplasm
AVESA.00001b.r3.6Dg0001063.1	6D:240522980:240526564	286	30255.97	7.66	6	Cytoplasm
AVESA.00001b.r3.6Dg0001793.1	6D:293766345:293768141	290	30787.64	8.61	6	Cytoplasm
AVESA.00001b.r3.7Ag0002628.1	7A:441960064:441961450	293	30764.73	8.24	6	Cytoplasm
AVESA.00001b.r3.7Cg0001277.1	7C:97629485:97630592	290	30525.47	9.39	5	Cytoplasm
AVESA.00001b.r3.7Cg0001881.1	7C:195084109:195087119	288	30486.66	8.53	6	Vacuoles
AVESA.00001b.r3.7Dg0000168.3	7D:10164408:10168502	300	32099.07	7.08	6	Endoplasmic reticulum
AVESA.00001b.r3.7Dg0001865.1	7D:376567968:376569553	247	25032.03	5.33	6	Vacuoles

### 3.2 Phylogenetic analysis of *A. sativa* AQP proteins

**Figure 1:**
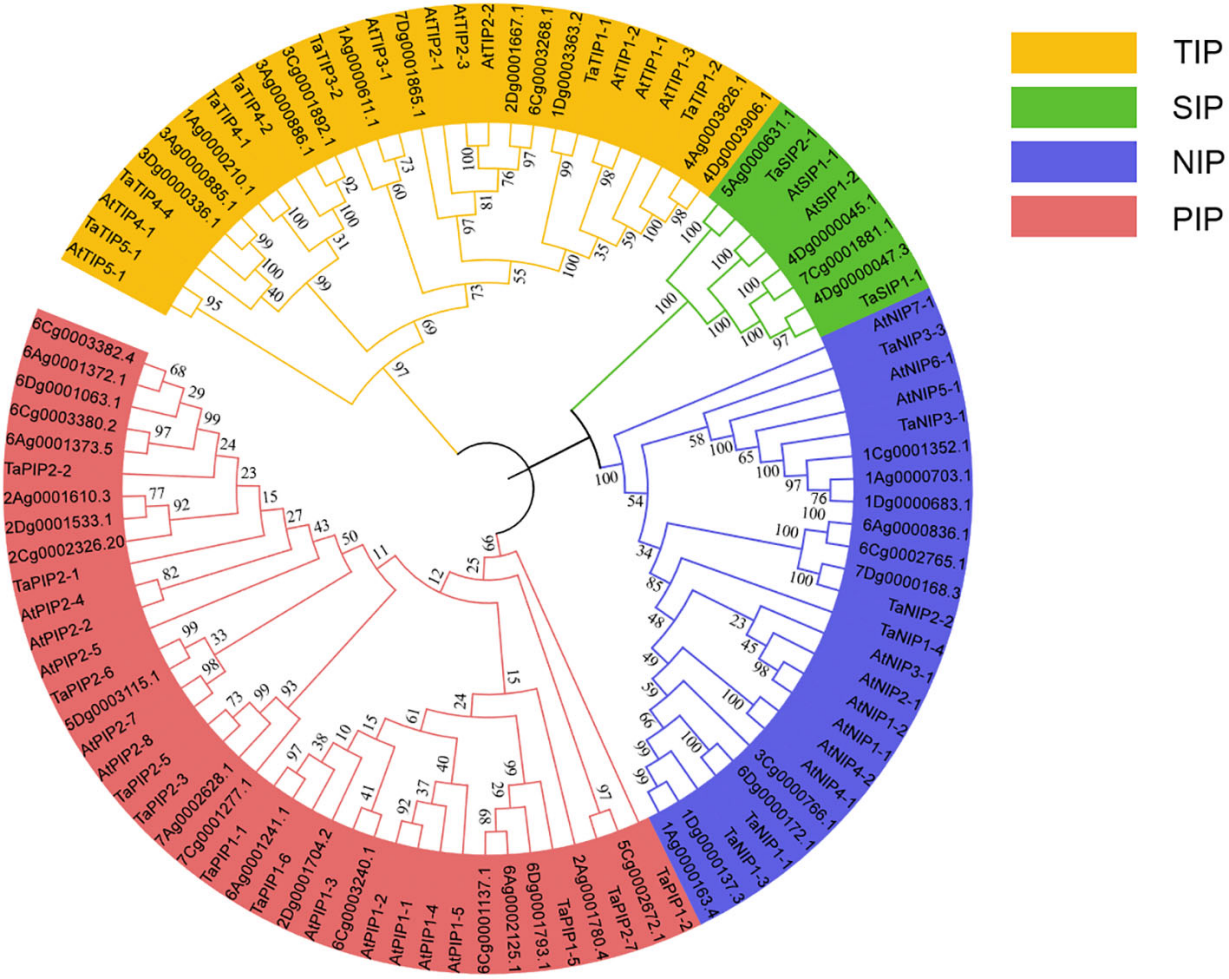
Phylogenetic tree of *A. sativa* AQP family members.

To further analyze the evolutionary relationship of AQP family genes and to mine the homologous evolutionary relationship between *A. sativa* and other species, this study constructed a phylogenetic tree used 25 protein sequences from *T. aestivum* 30 protein sequences from *A. thaliana* and 45 protein sequences from *A. sativa*. The *A. sativa* AQP proteins co-clustered into four subfamilies; 12 members of the TIP subfamily, 19 members of the PIP subfamily, 10 members of the NIP subfamily and 4 members of the SIP subfamily. The TIP, PIP, and NIP subfamilies had similar numbers of members whereas the SIP subfamily had substantially fewer members than the others, which was consistent with the distribution of members among the subfamilies of the *A. thaliana* AQP family (**Figure 1**).

### 3.3 Conserved motifs and gene structure of the *A. sativa* AQP gene family

**Figure 2:**
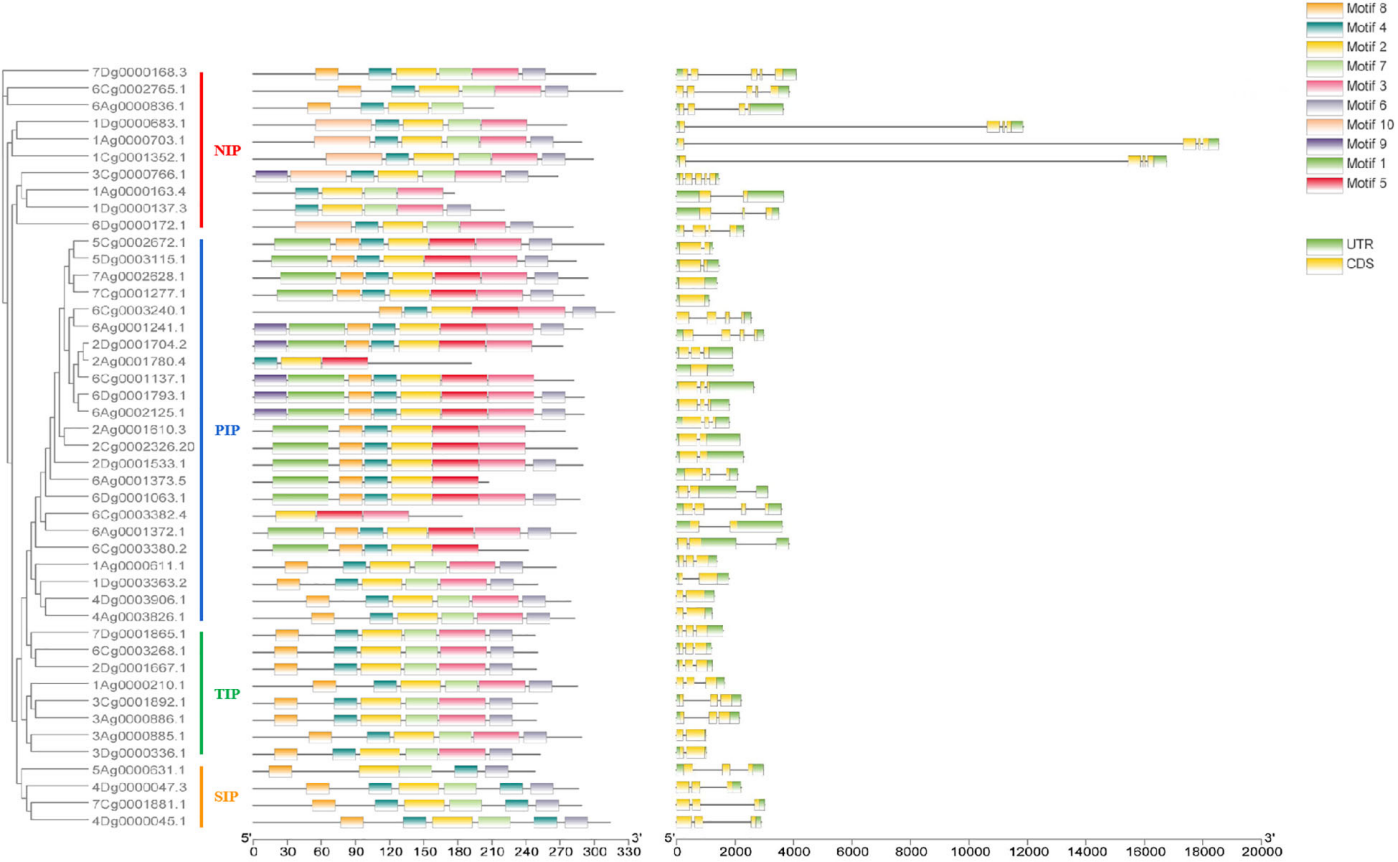
Conserved motifs and gene structure of AQP family genes.

To analyze the sequence conservation of AQP gene families, the conserved motifs of 45 AQP family members were identified by MEME, and a total of 10 motifs were selected and outputted. The results showed greater similarity in the types and numbers of conserved motifs among members within the same subfamily and the, frequencies of Motif 2, Motif 3, Motif 4, Motif 6 and Motif 8 were higher. Motif 2, Motif 4, Motif 6 and Motif 8 appeared more frequently, and 45 and 44 family members contained Motif 2 and Motif 4, respectively. In addition, high consistency of motifs was observed among the same subfamily, but some unique motifs were found among the different subfamilies, e.g., all members of the PIP subfamily had Motif 5, and Motif 5 was not observed in members of the other subfamilies. All five members of the NIP subfamily (1Dg0000683.1, 1Ag0000703.1, 1Cg0001352.1, 3Cg0000766.1 and 6Dg0000172.1) contain the unique Motif 10 (**Figure 2**). The position and type of motifs in the unified subfamily were found to be highly conserved, whereas specific motifs may exist in different subfamilies, which may reflect of the functional conservatism among subfamilies.

### 3.4 Promoter cis-acting elements of the *A. sativa* AQP gene family

**Figure 3:**
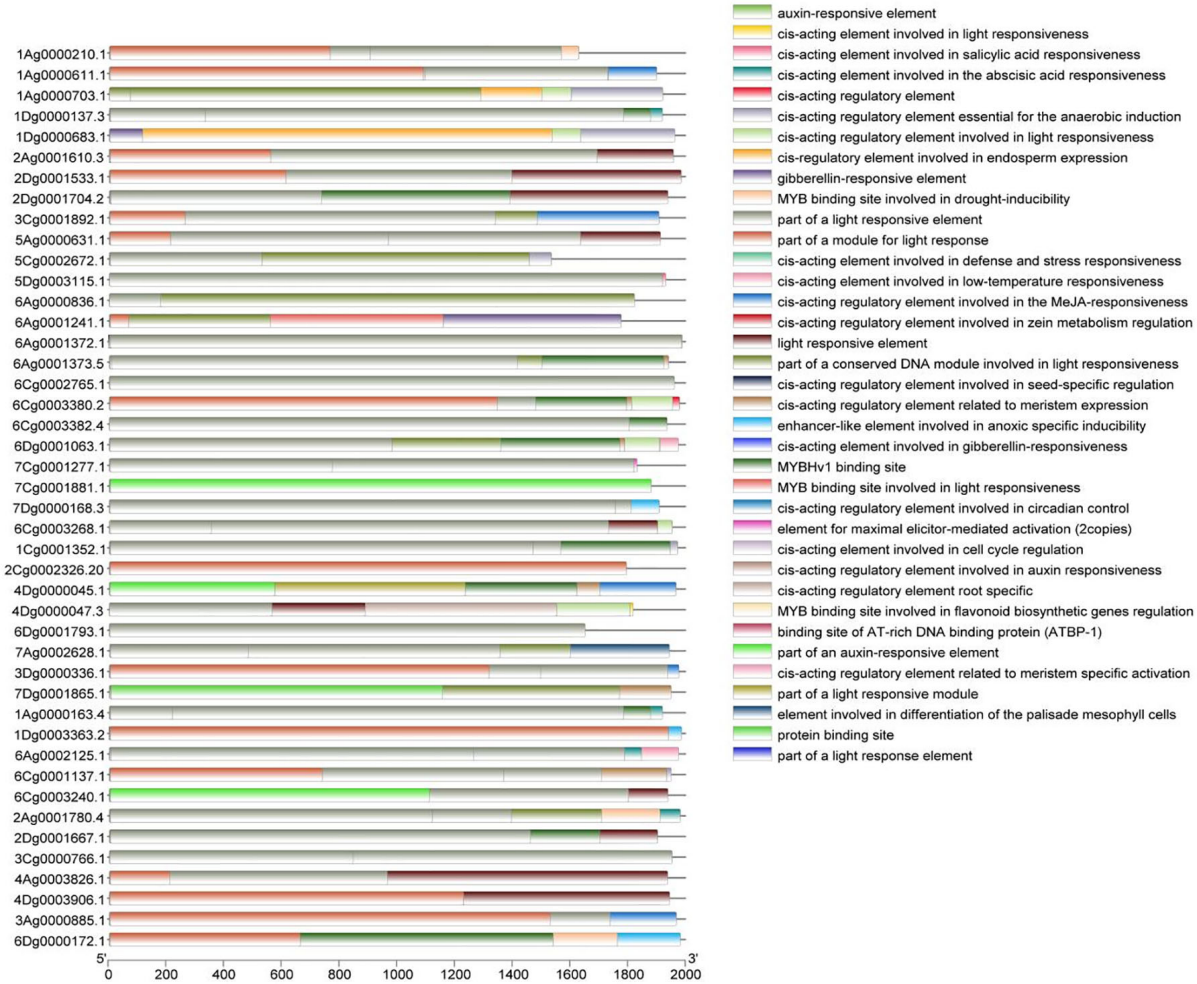
Cis-acting elements in the promoter region of the *A. sativa* AQP gene family.

To obtain a more in-depth understanding of the function of the *A. sativa* AQP gene family, the cis-acting elements located 2000 bp upstream of the promoters of the 45 members were analyzed (**Figure 3**). The identified cis-acting elements can be broadly categorized into light-response-related elements, abiotic stress-related elements, phytohormone-responsive elements, and growth-development-responsive elements, which suggests that the AQP gene family may play a role in the growth and development of *A. sativa* and in coping with a variety of environmental changes. Moreover, 44 family members contained light-responsive elements, and the AQP gene family also contained various phytohormone-responsive elements, such as the growth hormone-responsive element AuxRE, the salicylic acid-responsive element TCA-element, and the gibberellin-responsive element GARE-motif, etc. The AQP gene family was also identified as the most effective in the development of *A. sativa*. Several elements related to the responses to abiotic stresses, including the anaerobic response element ARE, and the low-temperature response element LTR were identified. In addition, several elements related to growth and development were also found.

### 3.5 Analysis of the chromosomal localization of AQP family genes in *A. sativa*

**Figure 4:**
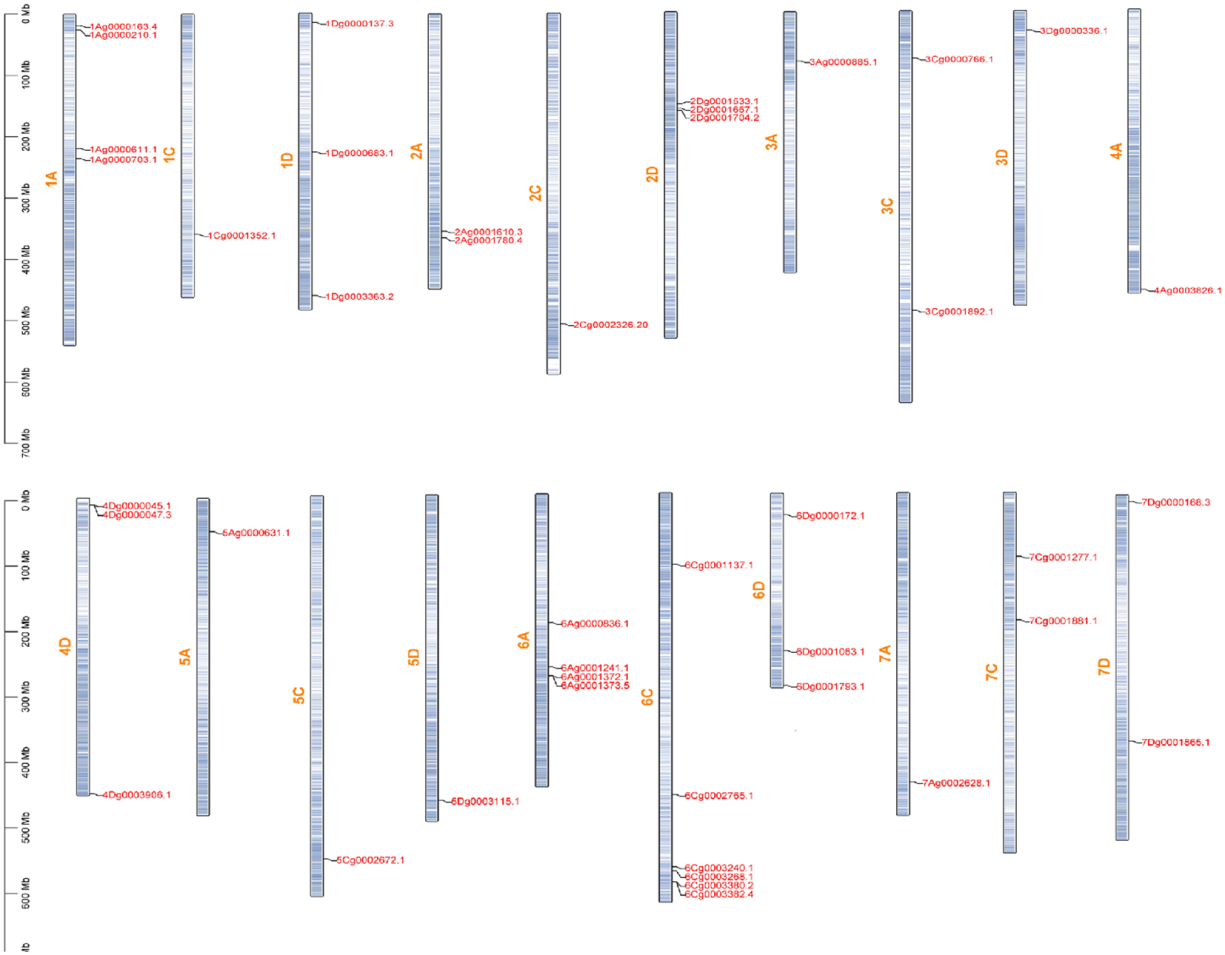
Chromosomal locations of *AsAQP* genes.

Based on the genomic information of *A. sativa*, the distribution of 45 A*. sativa* AQP genes on 21 chromosomes was analyzed (**Figure 4**). The results showed that these genes were unevenly distributed on 20 chromosomes and chromosome 4C did not contain an AQP gene. More than six genes were located on chromosome 6C. Moreover, the analysis revealed the presence of four genes on chromosomes 1A and 6A, three genes on chromosomes 1D, 2D, 4D, and 6D, two genes on chromosomes 2A, 3C, 7C, and 7D, and only one gene on chromosomes 1C, 2C, 3A, 3D, 4A, 5A, 5C, 5D, and 7A. AVESA.00001b.r3.3Ag0000886.1 is not shown because it was not found to be localized on a chromosome and could therefore not be plotted in the figure.

### 3.6 Gene duplication events and collinearity analysis of the *A. sativa* AQP gene family

**Figure 5:**
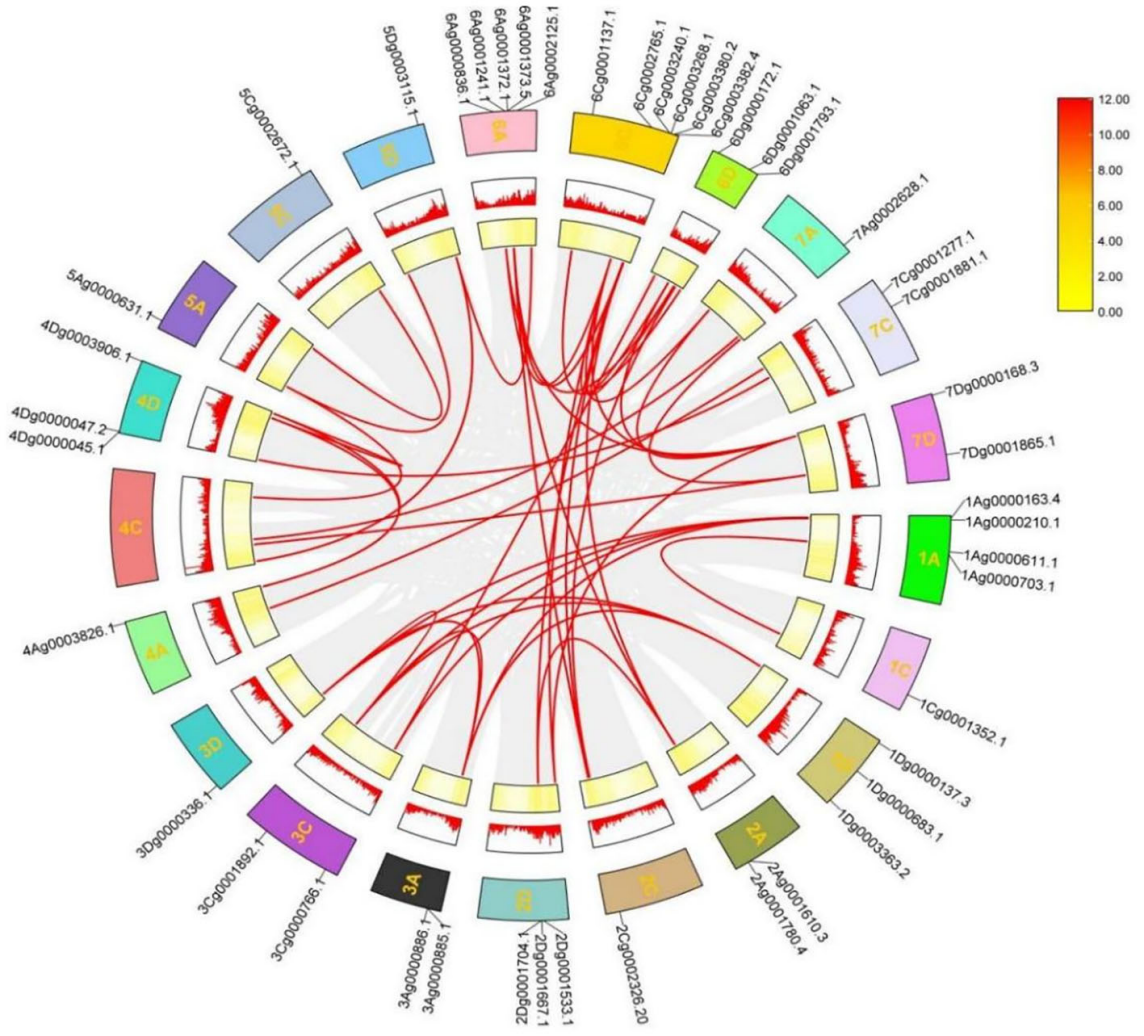
Chromosomal distribution and intragroup covariance analysis of the *AsAQP* genes. The gray section represents the collinearity of all genes within *A. sativa*; the red line represents the collinearity within the AQP gene family.

The phenomenon of gene duplication is widespread and plays an integral role in plant evolution. To analyze the evolutionary process among *A. sativa* AQP family genes, genome-wide duplication events among AQP family members were identified and analyzed and MCScan X software was used to draw Circos plots This analysis showed that a total of 24 genes formed genome-wide duplication pairs, that the 24 pairs of homologous genes were segmentally duplicated and that the duplication pairs resulted in many homologues of *AsAQP* genes among chromosomes, which increases the likelihood of evolution (**Figure 5**). These findings suggests that gene duplication events play an important role in the expansion of *AsAQP* genes.

**Figure 6:**
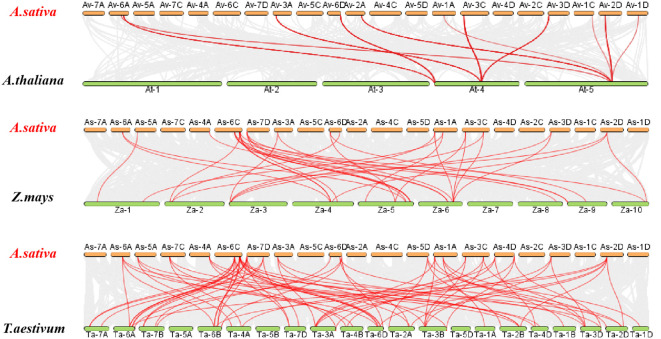
Analysis of AQP gene collinearity between *A. sativa* and three representative plants. The grey lines in the background indicate blocks of collinearity within *A. sativa* and the indicated plants, whereas the red lines indicate homozygous AQP gene pairs.

To further understand the possible evolutionary events of the AQP gene family in different crops, covariance analysis was performed on *A. sativa* simultaneously with *A.thaliana* and *T. aestivum* (**Figure 6**). The results revealed the existence of many collinear genes existed in *A. sativa*, *A. thaliana* and *T. aestivum*. Moreover, 4225 collinearity pairs were found between *A. sativa* and *A. thaliana*, which accounted for 4.59% of the total number of genes in *A. sativa.* In addition, 21 co-collinearity pairs were detected between *A. sativa* and *A. thaliana*, and 44,515 collinearity pairs were observed between *A. sativa* and *Z. mays*, which accounted for 43.01% of the total number of genes in *A. sativa.* Additionally, 35 collinearity pairs were found between *A. sativa* and *Z. mays*, and 45, 200 collinearity pairs were detected between *A. sativa* and *T. aestivum*, which accounted for 48.50% of the total number of genes in *A. sativa.* Seventy-six homologous gene pairs were also detected in *A. sativa* and *T.aestivum.*

### 3.7 Analysis of the gene regulatory network of the *A. sativa* AQP gene family

**Figure 7:**
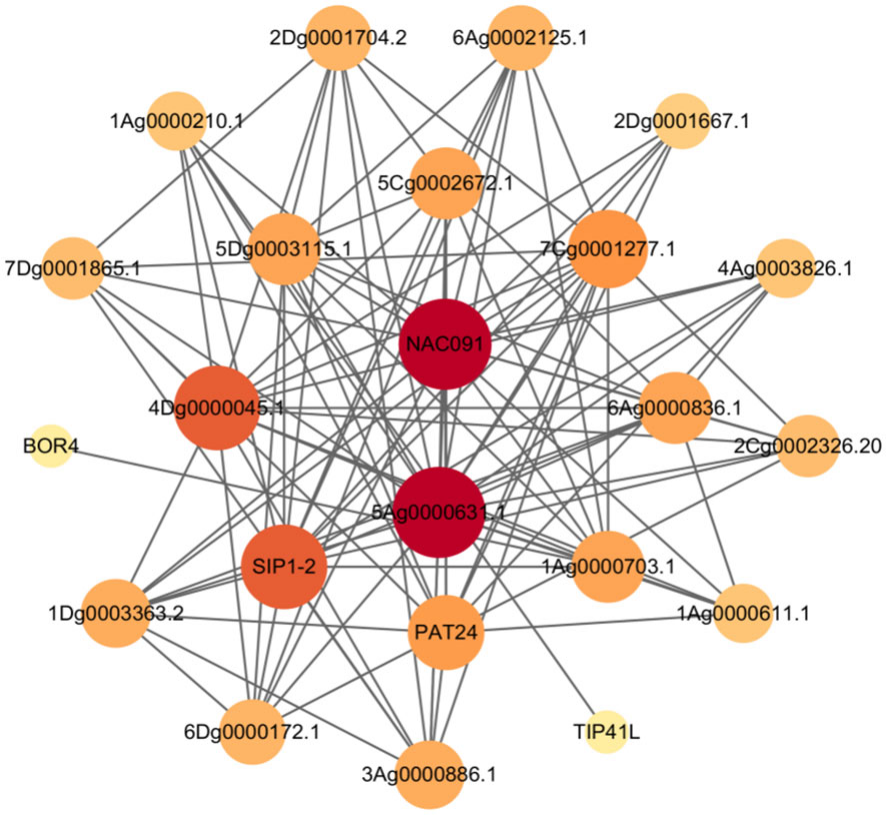
*A. sativa* AQP protein interaction network diagram.

To better understand the function of the *A. sativa* AQP gene family, 45 A*. sativa* AQP proteins were uploaded to the STRING online database and compared with the *A. thaliana* protein database as a reference. Construction of the interaction network of *A. sativa* AQP proteins (**Figure 7**) revealed 23 nodes (representing AsAQP proteins) and 102 edges (representing interactions between proteins) in the regulatory network. This network indicated that 23 of the 45 AsAQP had interaction relationships, and among these, 5Ag0000633.1 had interaction relationships with 16 AsAQP proteins, which was the largest number of interacting proteins. This finding suggests that 5Ag0000633.1 may be at the core of the AQP gene family.

### 3.8 Analysis of expression patterns of *AsAQP* genes in different *A. sativa* tissues

**Figure 8:**
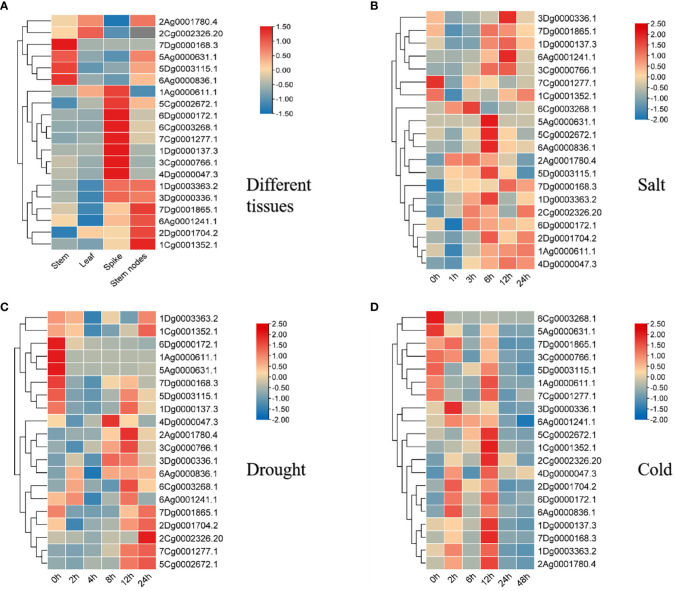
Expression heatmap of the *A. sativa* AQP gene family. **(A)** Tissue expression analysis of *AsAQP* genes in *A. sativa* **(B)** Expression of *AsAQP* genes under salt stress conditions. **(C)** Expression of *AsAQP* genes under drought stress conditions. **(D)** Expression of *AsAQP* genes under cold stress conditions.

Twenty genes were randomly selected from the four subfamilies of *A. sativa* AQP proteins (TIP, PIP, NIP and SIP), which contained different protein domains and cis-elements, and their tissue-specific expression was studied. The expression heatmap of *A. sativa* AQP family genes in different tissues was drawn (**Figure 8A**). The results showed that 20 genes of different AsAQP subfamilies showed differential expressed in different organs of *A. sativa*, and the expression levels of most TIP subfamily genes, such as 7Dg0001865.1, 1Dg0003363.2, and 3Dg0000336, were higher in the stem nodes and spikes than in the stems and leaves. The expression of 1Ag0000611.1 was higher in spike and leaf. The expression of PIP subfamily genes was higher in stem nodes than in other tissues. Most of the NIP subfamily genes with the exception of 7Dg0000168.3 and 6Ag0000836.1 exhibited higher expression in spikes. Among the SIP subfamily genes, 4Dg0000047.3 and 5Ag0000631.1 were expressed at higher levels in stems, spikes and stem nodes. The expression levels of most genes were higher in the spikes and stems of *A. sativa* but lower in leaves and stems, which may be closely related to their functions and participation in *A. sativa* growth and development at different stages.

### 3.9 Response and expression pattern of *A. sativa* AQP family genes under salt, drought and cold stress

To understand the response of *AsAQP* gene to abiotic stress, we further analyzed the expression patterns of 20 members of the AQP gene family under three abiotic stresses (salt, cold and drought). The results presented in **Figure 8B** show that 20 *AsAQP* genes exhibit varying degrees of response to the three stress treatments, and 6Cg0003268.1 was significantly upregulated after 3 h of NaCl treatment. 2Dg0001741.2, 1Dg0003363.2, 5Dg000311.1, 6Ag0000836.1, 5Cg0002672.1, and 5Ag0000631.1 were significantly upregulated and peaked after 6 h of NaCl treatment. In addition, several AQP genes were upregulated after 12 h of NaCl treatment, indicating that some AQP genes involved in the response of *A. sativa* to salt stress. A cluster analysis showed that *AsAQP* genes located in the same evolutionary branch exhibit certain similarities in response to NaCl treatment. For example, 7Dg0001865.1 and 1Dg0000137.3 showed similar expression patterns after NaCl treatment, and their expression levels were upregulated after the same treatment duration. However, 2Ag0001780.4 and 5Dg0003115.1, which are located in the same evolutionary branch, were exceptions: the expression of 2Ag0001780.4 was significantly upregulated after 1 h of NaCl treatment and then decreased, whereas the expression of 5Dg0003115.1 was significantly upregulated after 6 h of NaCl treatment. These results indicate that the 2Ag0001780.4 and 5Dg00031151.1 genes may play a significant role in the response to NaCl stress but play different roles at different times, and their functions may differ, suggesting that these genes may respond to NaCl stress through interaction. The results presented in **Figure 8C** showed that the expression levels of 7Dg0001865.1, 7Cg0001277.1, 2Cg0002326.20, 2Dg0001704.2, 7Dg0001865.1 and 1Cg0001352.1 were upregulated and peaked after PEG treatment for 24 h. The expression levels of 4Dg0000047.3, 2Ag0001788.4 and 2Cg0002326.20 were significantly upregulated after PEG treatment for different durations, suggesting that some AQP genes were related to the response of *A. sativa* to drought stress. As shown in **Figure 8D**, the expression levels of 3Dg0000336.1, 1Cg0001352.1, 6Ag0000836.1, 4Dg0000047.3 and 2Cg0002326.20 were upregulated after low temperature treatment at 4°C for different durations, indicating that the expression of some AQP genes was induced by low-temperature stress. Among these genes, 1Cg0001352.1 was the most highly induced gene. 6Cg0003268.1 was inhibited under low-temperature stress, and the expression levels of the remaining 19 AsAQP genes were not inhibited with increases in the stress duration, moreover, the expression levels of most genes peaked after 12 h under low-temperature stress.

## 4 Discussion

With the rapid development of genome sequencing technology, an increasing number of plant genomes have been successfully sequenced, which has facilitated the identification of a wide range of plant genomes (Rose et al., 2021). The sequencing of the *A. sativa* genome has been completed, and its genomic data are online, which facilitates studies of the *A. sativa* gene family and gene evolution (Peng et al., 2022). Water is the basis of plant life, and water molecules are able to enter the cell from the environment through the cell membrane and participate in life activities. Seventy percent of transmembrane transport is accomplished through specific membrane proteins known as AQP proteins, and the AQP genes involved in the regulation of plant growth and development are abundant, diverse, and widely distributed in the plant cells (Wang and Cui, 2016). Plant AQP proteins are intrinsic membrane proteins belonging to the MIP superfamily that play important roles in water transport across membranes, cellular osmoregulation, seed germination, lateral root production, carbon fixation, nutrient uptake and stress resistance (Chen et al., 2023). AQP are widely distributed in plant genomes, and genome-wide analyses of AQP in a variety of higher plants have been reported. However, studies on analyses of the functions of the *A. sativa* AQP family have not been conducted. Therefore, a genome-wide analysis of AQP in *A. sativa* is of great importance.

In this study, 45 AsAQP family members were isolated and identified from the *A. sativa* genome. This number of its gene family members is greater than that the numbers found in *A. thaliana, O. sativa*, and *Z. mays* (Chaumont et al., 2001; Johanson et al., 2001; Sakurai et al., 2005), which demonstrates the diversity of these genes among different species, and it is hypothesized that gene duplication and functional differentiation of AQP genes occured during the process of plant evolution (Dassanayake et al., 2011). Based on these findings combined with the evolutionary tree of the AQP protein family, all AsAQP can be divided into four subfamilies (PIP;TIP;NIP and SIP), which is consistent with the AQP delineation categories of AQP that have been previously studied by researchers (Mahnaz and Parviz, 2023). Due to the highly compartmentalized nature of plant cells, the subcellular localization of aquaporins (AQP) exhibits diversity. AQP are present in all cellular and subcellular compartments of plants (Zhang et al., 2017). The AsAQP family consists of four subfamilies with different subcellular localizations, including the endoplasmic reticulum, vacuoles, and cytoplasm. The different subfamily members are localized to different positions in the cell, and this localization is likely closely associated with the molecular functions of the proteins. However, this study found that the *A. sativa* PIP subfamily member 2Ag0001780.4 is localized to the endoplasmic reticulum, while the other PIP subfamily members are localized to the cytoplasm. In *A. thaliana*, the NIP subfamily member NIP5;1 is localized to the plasma membrane, while another subfamily member, NIP2;1, is localized to the endoplasmic reticulum (Mizutani et al., 2006; Takano et al., 2006). This is similar to the results of this study, where members of the same subfamily exhibit different subcellular localizations. In addition, the study also found diverse localizations among the TIP subfamily members. In *A. sativa*, seven TIP subfamily members, such as 4Ag0003826.1, 4Dg0003906.1, 1Ag0000611.1, *3Cg0001892.1*, *3Ag0000885.1*, 1Ag0000210.1 and 1Dg0003363.2, are localized to the cytoplasm, while five members, such as 3Dg0000336.1, 3Ag0000886.1, 7Dg0001865.1, *6Cg0003268.1* and 2Dg0001667.1, are localized to vacuoles. The subcellular localization of ScTIP1;1 in the grass species Stipa capillacea Keng is on the Vacuoles, and its gene expression can be induced by salt, drought, and low temperature stress (Dong et al., 2016). Similarly, all PeTIPs in bamboo are localized on the Vacuoles, and RNA-seq data and qPCR results confirm their involvement in response to low temperature, drought, and strong light stress (Zhu et al., 2021). Based on this, it can be speculated that members of the AsTIPs subfamily in *A. sativa*, such as 3Dg0000336.1, 3Ag0000886.1, 7Dg0001865.1, 6Cg0003268.1, and 2Dg0001667.1, play different roles in combating external stressors, thereby regulating plant tolerance to abiotic stresses such as drought, salt stress, and cold.

In the present protein interaction regulatory network of the *AsAQP* gene family, NIP family members exhibit strong protein interactions with other AQP subfamilies, especially the SIP and PIP subfamilies. The NIP subfamily member 1Ag0000703.1 also interacts with a boron transporter (BOR4), which is speculated to be involved in the transport of boric acid and other substances. Boric acid is an essential trace element in pollen tube development, fertilization and other reproductive processes (Masataka et al., 2014), TIP subfamily members, some members of the PIP family and a palmitoyl transferase protein (PAT24) interact with palmitoyl transferase proteins involved in protein palmitoylation, regulation of cellulose biosynthesis processes, and regulation of secondary cell wall biogenesis (Chen et al., 2021). TIP, NIP, and PIP family members also interact with NAC091, which belongs to the NAC family of transcription factors, a superfamily of plant-specific transcription factors. *A. thaliana* NAC091 is a transcription factor that interacts with turnip crinkle virus (TCV) capsid protein and is involved in regulation of the *A. thaliana* defense response to TCV (Tao et al., 2005; Teresa et al., 2014). NAC091 is also upregulated by NaCl, low temperature (4°C) and ABA, among other stresses, and can also transfer ER stress signals from the plasma membrane to the nucleus; thus, this transcription factor plays an important role in alleviating ER stress and promoting cell survival (Jian, 2020). It can be inferred that some members of the *A. sativa* AQP family interact with the transcription factor NAC091 to play an important role in the regulation of abiotic stress in forage plants.

In an environment with an abiotic stress, such as salt, drought and low temperature, dry air, ion stress and temperature changes in the surrounding environment will cause water absorption difficulties in plants. The signal molecules generated by water loss enable plants to maintain their water balance by changing the expression of AQP (Sutka et al., 2011). An analyze of the expression patterns of AsAQP family genes under low temperature, salt and drought stress indicated that most AsAQP genes may be involved in the abiotic stress response. Low temperature stress upregulates the expression of 15 AsAQP family genes, which suggests that these genes may play a positive regulatory role in the molecular regulatory pathways that enhance the low-temperature adaptability of *A. sativa*, and these genes may be important candidate genes involved in the response of *A. sativa* to cold stress. GhTIP1;1 in cotton also exhibits an obvious positive regulatory effect, and upregulation of this gene in *A. thaliana* significantly increases the expression level of cold-response genes (Gongmin et al., 2022). Moreover, OsPIP2;7 overexpression in rice improves the survival rate of rice under low-temperature stress and affects the expression levels of other AQP genes in rice (Li et al., 2008). Salt stress significantly upregulated the expressions of 1Dg0003363.2, 2Ag0001780.4 and 6Ag0000836.1, which predicted that these three genes could positively regulate the salt tolerance of *A. sativa*, indicating that the positive regulatory function of AQP gene under salt and cold stress is relatively common. Drought stress inhibited the expression of 11 AsAQP family genes, which suggests that these genes may play a negative regulatory role in the molecular pathways that enhance drought adaptability in *A. sativa*. The AQP gene expression patterns of *A. thaliana* were substantially different under drought stress; for example, AtPIP2;1 and AtPIP2;2 expression was down-regulated under drought stress (Jin, 2015), but AtPIP1;3 transcription was also detected; and AtPIPs expression was up-regulated in a few groups (Alexandersson et al., 2010). The analysis of OsPIP1 expression in rice under drought stress revealed that the expression of these two genes was up-regulated, whereas the expression of all OsPIP2s genes were down-regulated (Liu et al., 2006). These results indicate that the AQP gene expression patterns in plants under drought stress are complex. Similar to the above-described results, this study found that AsAQP genes in *A. sativa* under drought stress mainly exhibit two expression patterns: an “up-down-regulated” expression pattern, and a “down-up-regulated” expression pattern. These results indicate that the expression of AQP gene expression in plants under drought stress is regulated by a complex signaling network, and the regulatory mechanism of AQP genes in plant drought resistance needs further study. 6Cg0003268.1 and 3Dg0000336.1 belong to the NIP subfamily types and show similar expression patterns under drought and salt stress, which further indicates that the evolutionarily similar genes exhibit functional redundancy to a certain extent. Moreover, the same type of gene also shows functional differentiation, and low-temperature treatment strongly inhibited the expression of 6Cg0003268.1 but only slightly change the expression level of 1Ag0000611.1. The expression of 6Ag0000836.1 was significantly up-regulated under salt, drought and cold stress, which suggests that this gene is an important candidate gene involved in the biotic stress response. *A. sativa* is an important and high-quality forage that is sensitive to low temperature, drought and high salt (Song et al., 2013; Kamal et al., 2022). Further study of *AsAQP* gene functions in the abiotic stress response is of great significance for the germplasm conservation and molecular assisted breeding of *A. sativa.*

## 5 Conclusions

In this study, the AQP gene family in the whole genome of *A. sativa* was analyzed, and a total of 45 AQP genes were identified through analyses of the evolutionary relationships, gene structure, gene duplication, promoter and regulatory network of the AQP gene family based on bioinformatic data. According to the *A. thaliana* AQP subfamily classification, the AQP gene family is divided into 4 subfamilies, and the PIP and SIP subfamilies have the highest (19) and fewest (4) members. AQP genes from the same evolutionary branch were found to have similar genes and structures. Among the 45 AsAQP, 23 proteins had interaction relationships, and 5Ag0000631.1 had the largest number of interacting proteins. An expression analyses revealed that these 20 genes were expressed in all tissues, and the expression of these genes showed marked differences among different tissues and organs, indicating that these *AsAQP* genes have different functions in the growth and development of *A. sativa*. The expression analysis of 20 *AsAQP* genes under cold, drought and salt stress showed that the expression of 6Ag0000836.1 gene of the NIP subfamily was significantly upregulated under different abiotic stresses, and this gene could be further verified as a key candidate gene involved in the responses to stress. The results of this study provide the complete members of the AsAQP protein family and their sequence characteristics and can serve as a reference and theoretical basis for further analyses of the structural characteristics of AsAQP and improving the stress resistance of *A. sativa*.

## Data availability statement

The oat data presented in the study are deposited in the Ensembl Plants website repository, accession number GCA_022788535”; Arabidopsis thaliana data presented in the study are deposited in the TAIR database repository.

## Author contributions

XZ: Conceptualization, Data curation, Formal Analysis, Visualization, Writing- original draft, Writing-review & editing. DY: Writing-original draft. LM: Investigation,Writing-review & editing. XW: Validation, Visualization, Writing-review & editing.
