# Supplementary figures and images for: Genome-wide analysis and expression of the aquaporin gene family in Avena sativa L
Source: Front Plant Sci. 2024 Jan 19;14:1305299. doi: 10.3389/fpls.2023.1305299 (PMC10836146; doi:10.3389/fpls.2023.1305299)

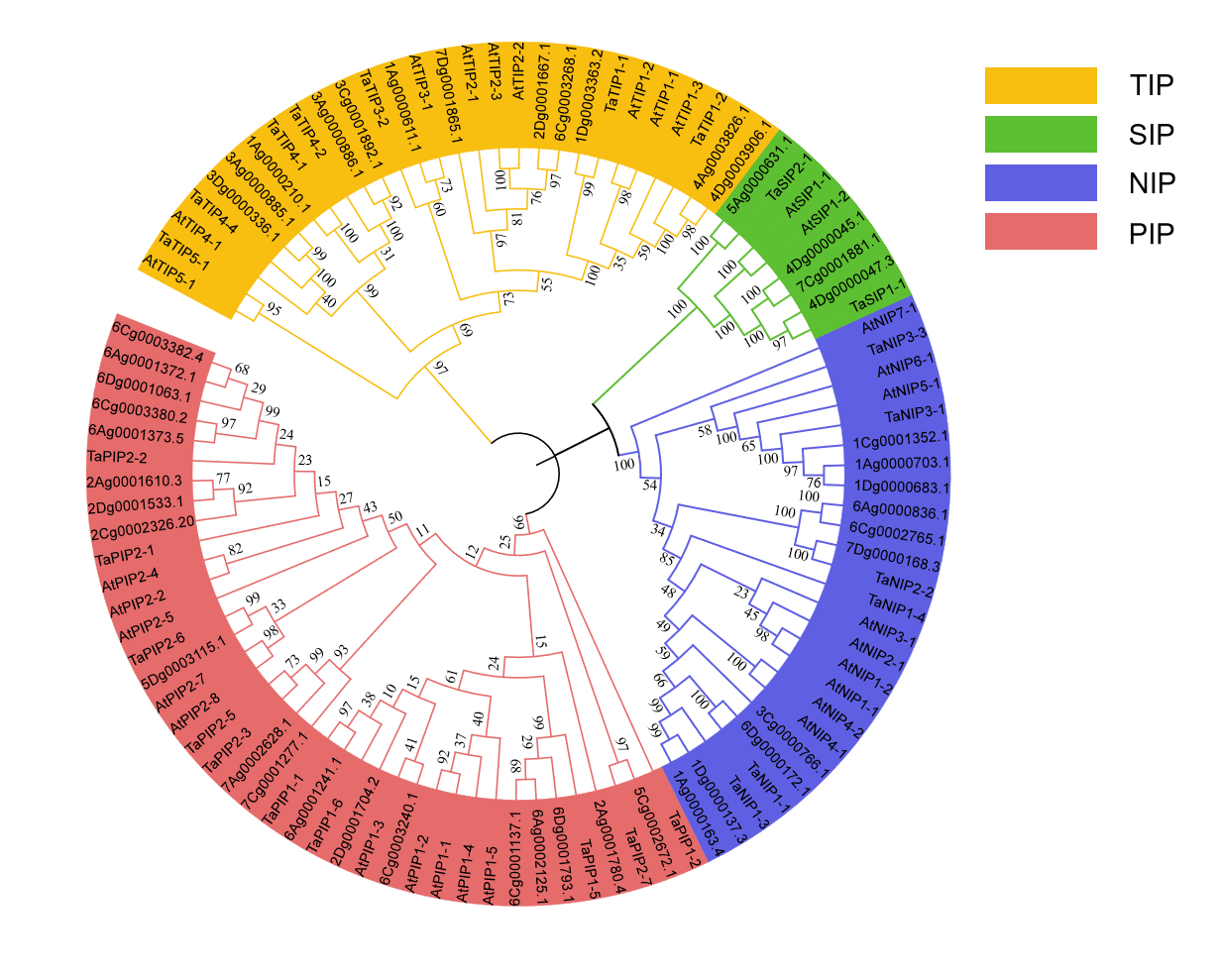

Supplement: Supplementary file 1 [file DataSheet_1.zip › Figure/Fig 1.Phylogenetic tree of A. sativa AQP family members..tif]

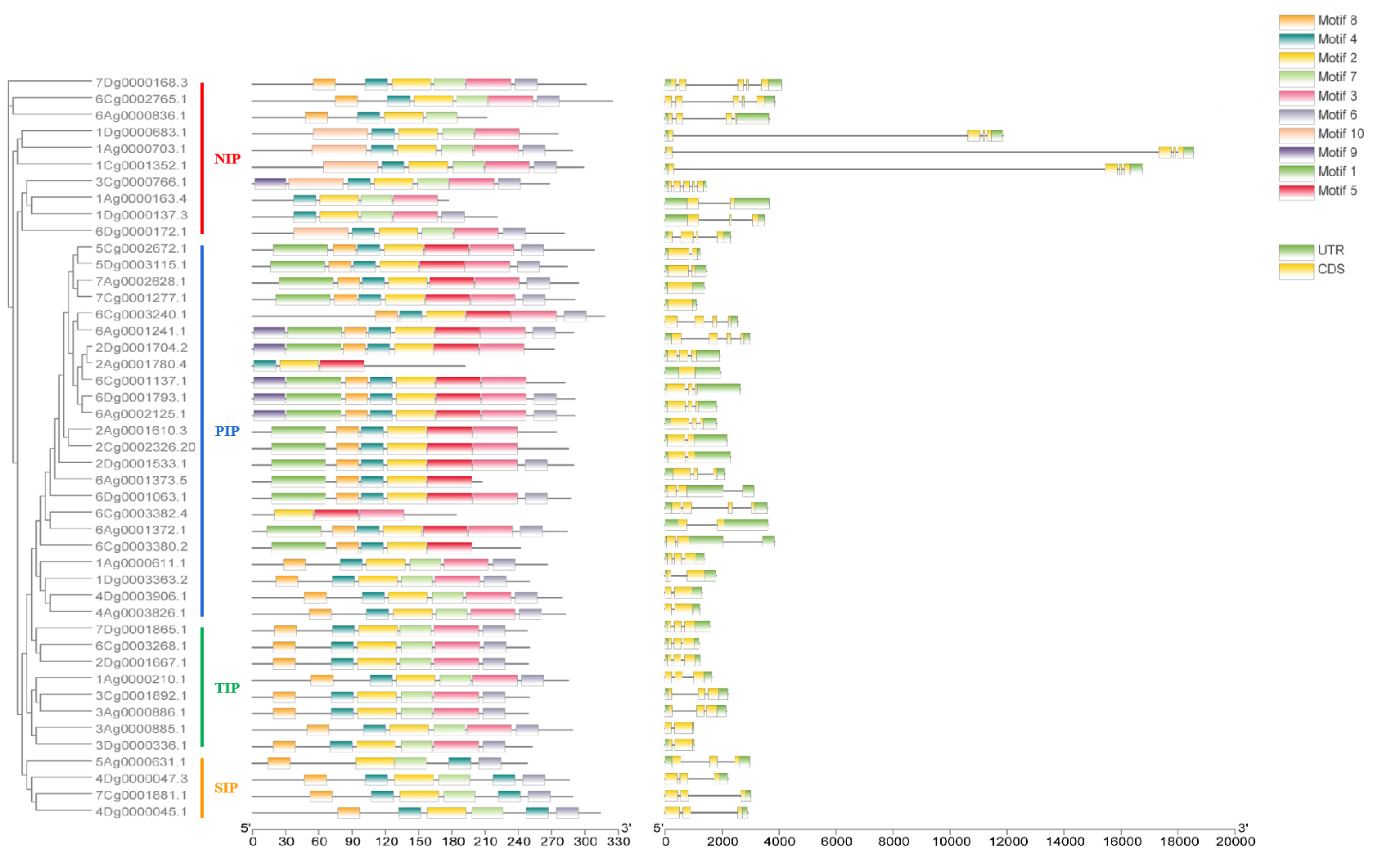

Supplement: Supplementary file 1 [file DataSheet_1.zip › Figure/Fig 2.Conserved motifs and gene structure of AQP family genes.tif]

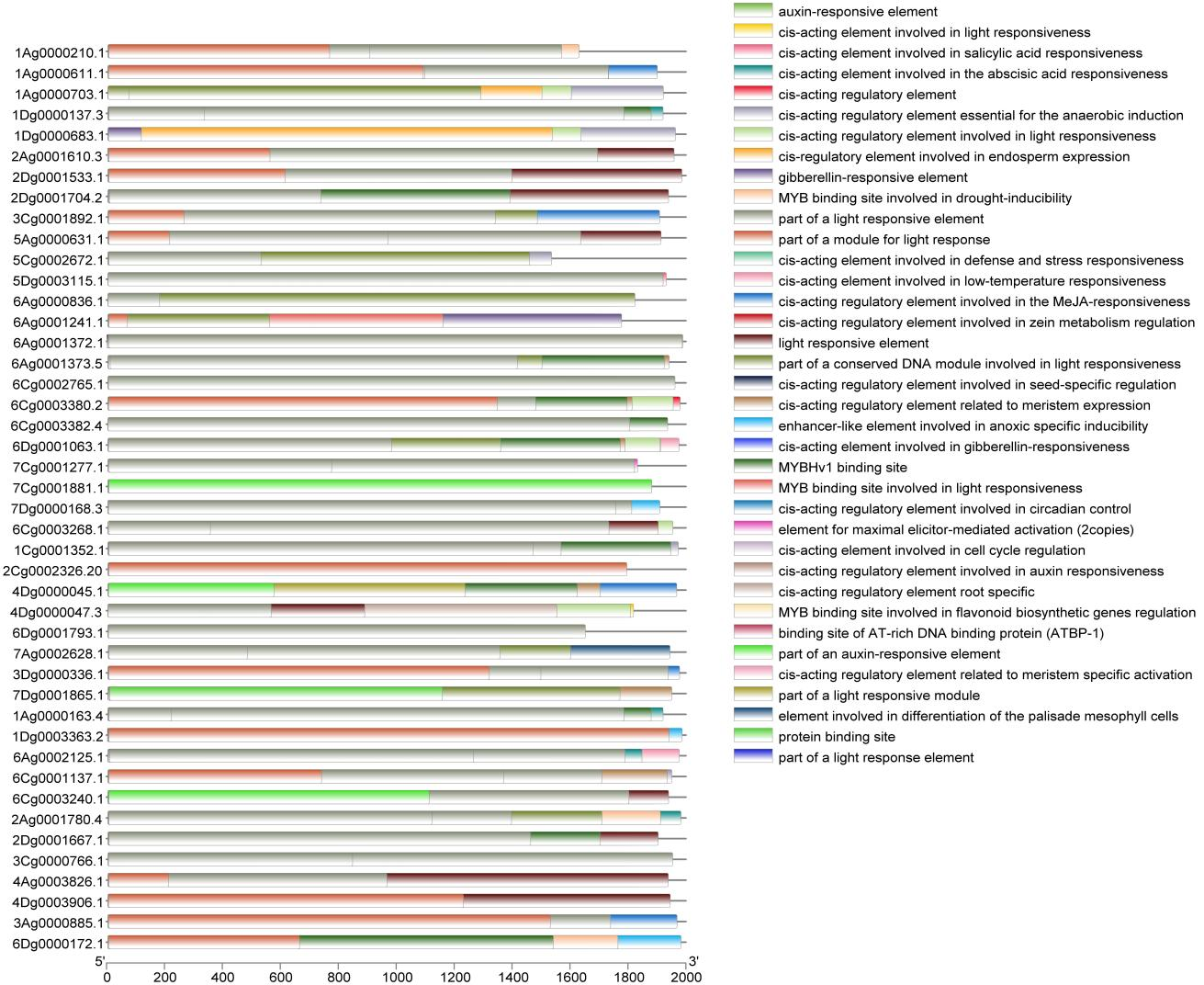

Supplement: Supplementary file 1 [file DataSheet_1.zip › Figure/Fig 3.Cis-acting elements in the promoter region of the A. sativa AQP gene family.tif]

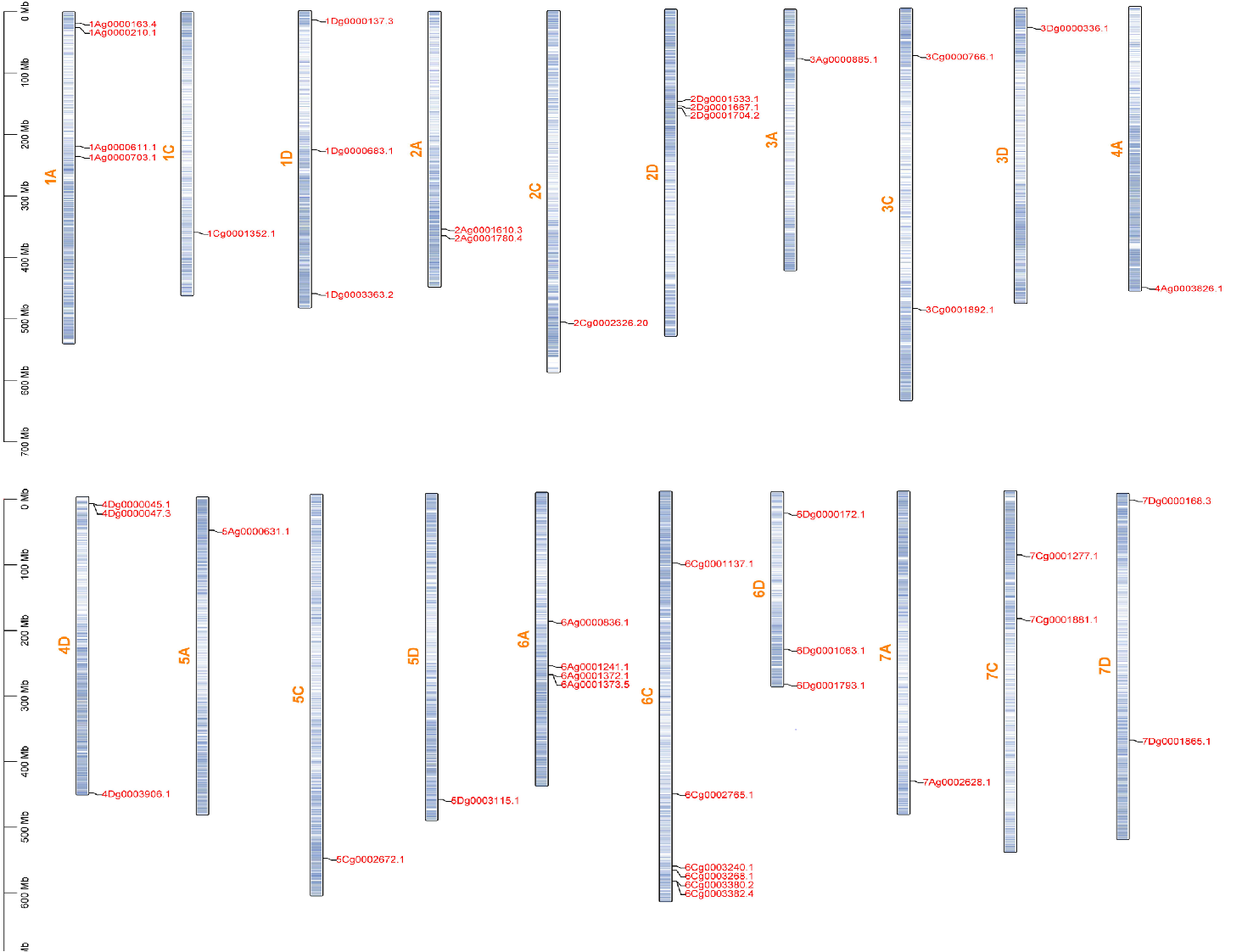

Supplement: Supplementary file 1 [file DataSheet_1.zip › Figure/Fig 4. Chromosomal locations of AsAQP genes..tif]

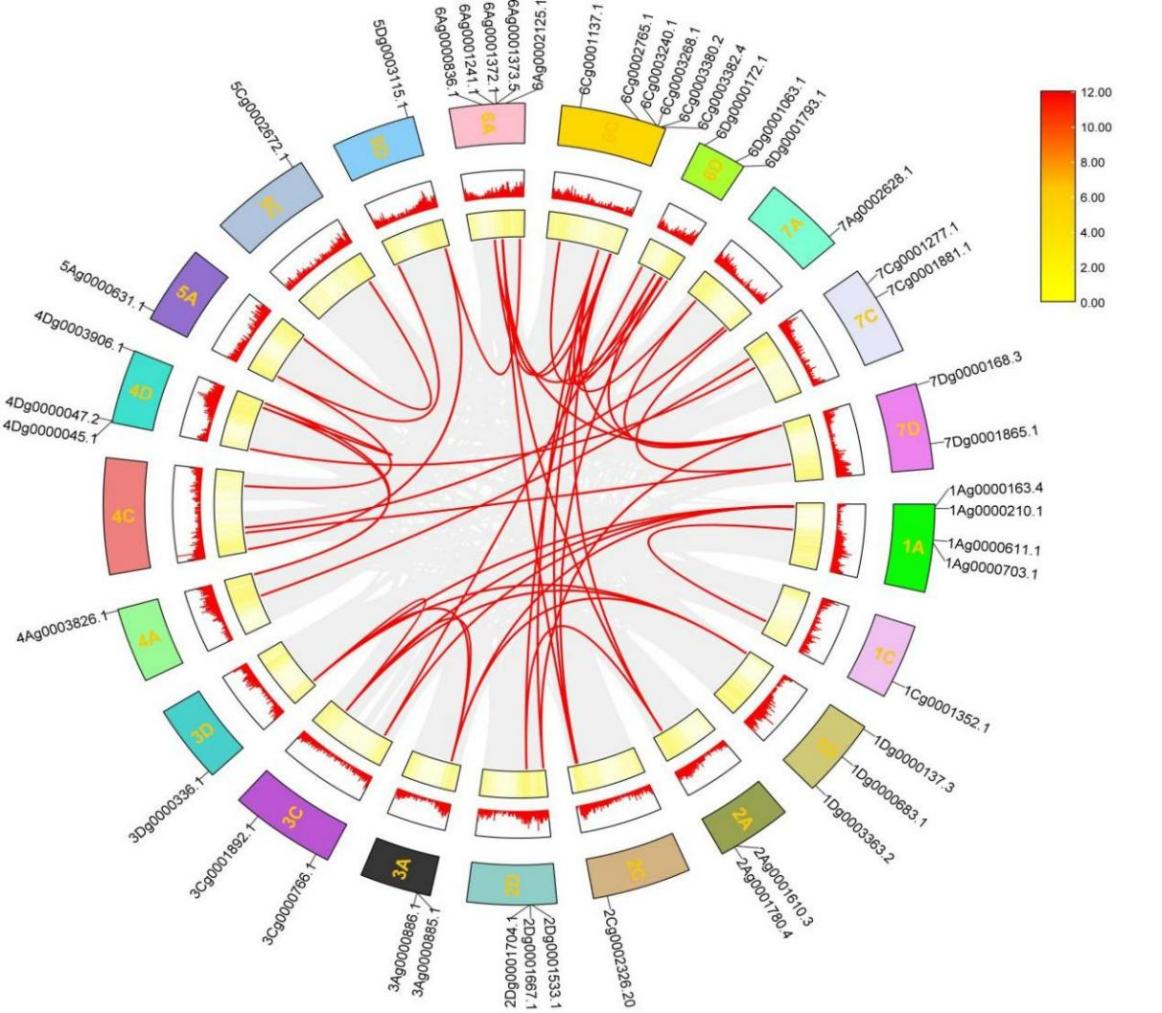

Supplement: Supplementary file 1 [file DataSheet_1.zip › Figure/Fig 5. Chromosomal distribution and intragroup covariance analysis of the AsAQP genes..tif]

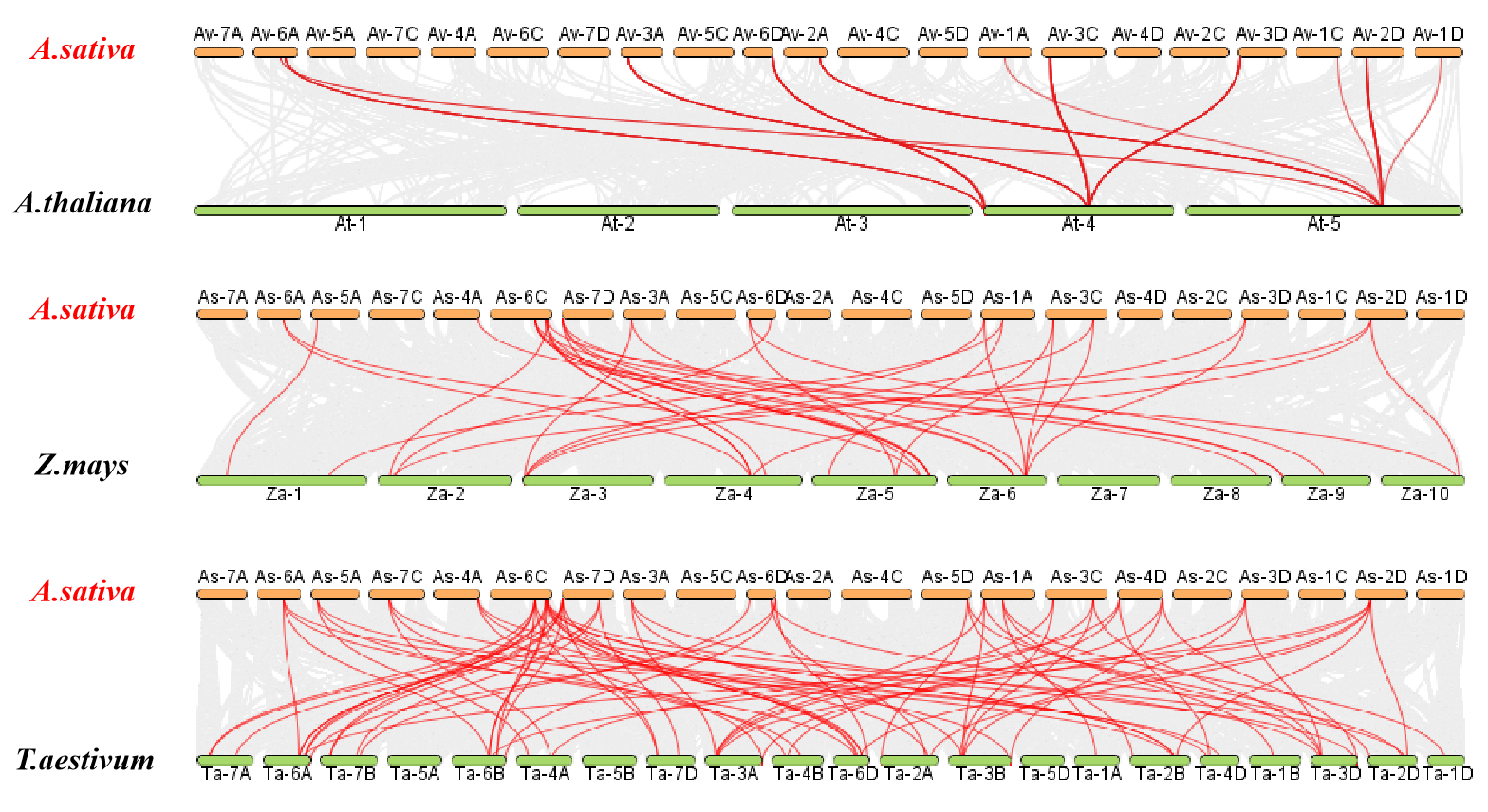

Supplement: Supplementary file 1 [file DataSheet_1.zip › Figure/Fig 6. Analysis of AQP gene collinearity between A. sativa and three representative plants..tif]

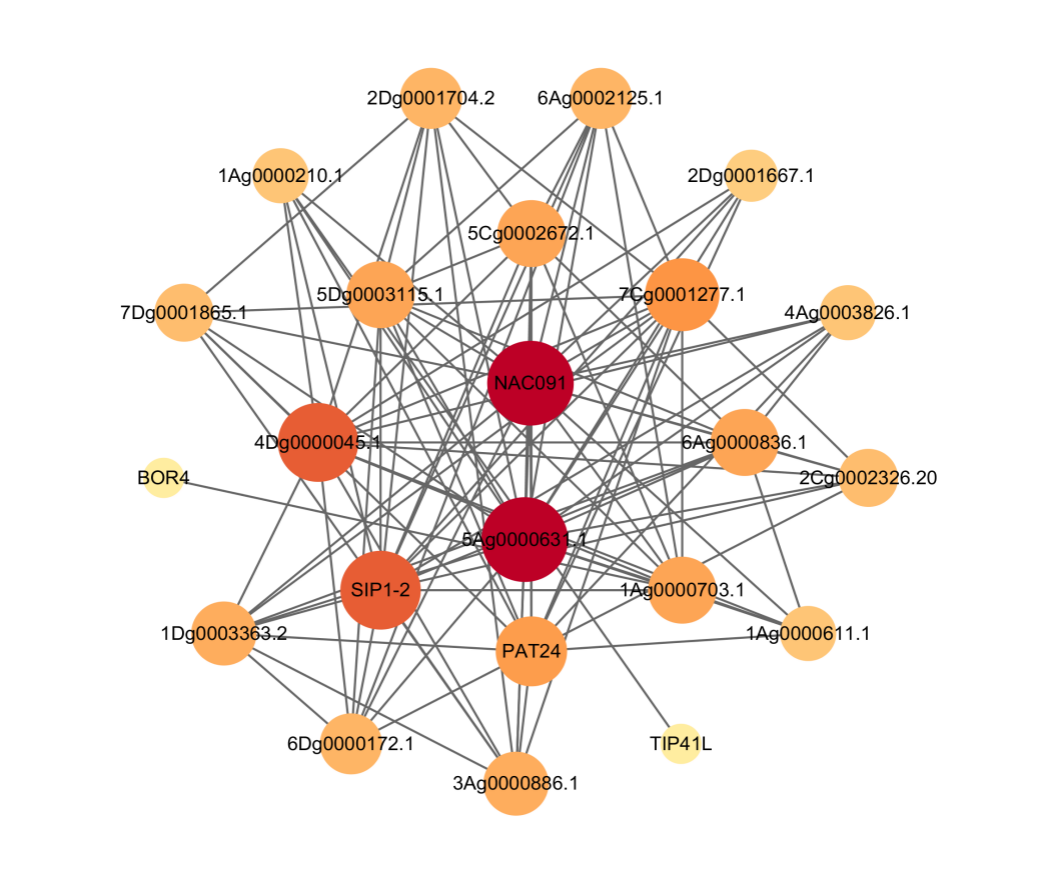

Supplement: Supplementary file 1 [file DataSheet_1.zip › Figure/Fig 7. A. sativa AQP protein interaction network diagram..tif]

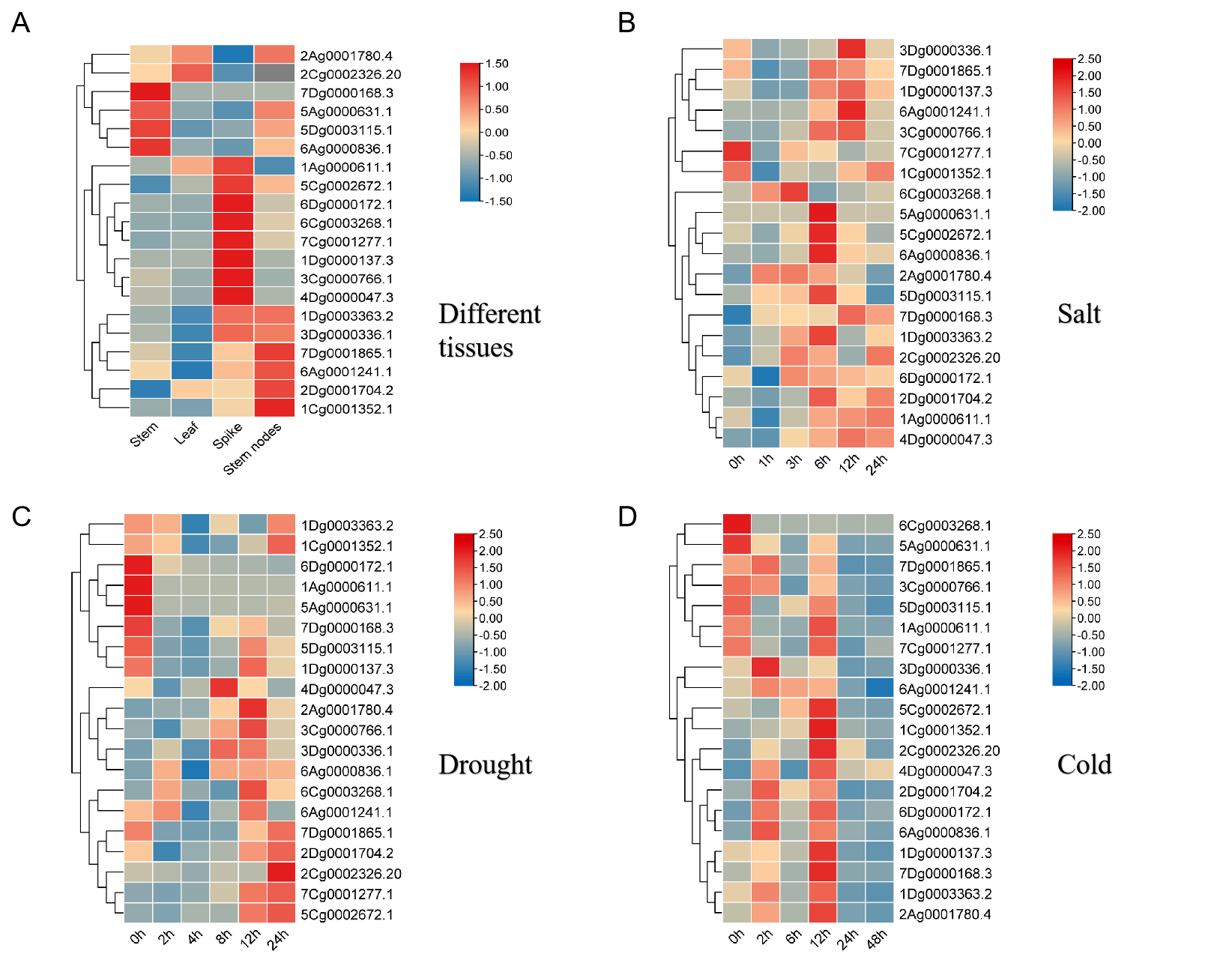

Supplement: Supplementary file 1 [file DataSheet_1.zip › Figure/Fig. 8 Expression heatmap of the A. sativa AQP gene family..tif]
